# High SOX2 Levels Predict Better Outcome in Non-Small Cell Lung Carcinomas

**DOI:** 10.1371/journal.pone.0061427

**Published:** 2013-04-19

**Authors:** Vamsidhar Velcheti, Kurt Schalper, Xiaopan Yao, Huan Cheng, Mehmet Kocoglu, Kavita Dhodapkar, Yanhong Deng, Scott Gettinger, David L. Rimm

**Affiliations:** 1 Department of Medical Oncology, Yale University School of Medicine, New Haven, Connecticut, United States of America; 2 Department of Pathology, Yale University, New Haven, Connecticut, United States of America; 3 Yale Center for Applied Biostatistics, New Haven, Connecticut, United States of America; H. Lee Moffitt Cancer Center & Research Institute, United States of America

## Abstract

**Background:**

SOX2 is an embryonic developmental transcription factor, which is important in the development of the respiratory tract. SOX2 overexpression is associated with aggressive disease in several tumor types. However, SOX2 overexpression and gene amplification associates with favorable outcome in lung squamous cell carcinomas (SCC) and dissimilar results have been reported in lung adenocarcinomas (ADC). The aim of the present study was to evaluate SOX2 expression in NSCLC and determine the relationship with clinico-pathological variables and outcome.

**Methods:**

SOX2 protein levels were measured in tissue microarrays (TMAs) containing FFPE samples from two independent lung cancer cohorts (n = 340 & 307) using automated quantitative immunofluorescence (QIF). Assay validation was performed using FFPE preparations of cell lines with known SOX2 expression. Associations of SOX2 levels with main clinico-pathological characteristics and with overall survival were studied using uni-and multivariate analysis.

**Results:**

SOX2 levels were higher in patients with SCC than in ADC in both cohorts (p value<0.0001). In the training cohort, NSCLC patients whose tumors showed high SOX2 (n = 245) had longer survival than those with low SOX2 levels (log rank p = 0.0002). Comparable results were observed in the second independent validation cohort, log rank p = 0.0113. SOX2 positive cases showed a 58% reduction in risk of death in Cox univariate analysis (hazards ratio-HR = 0.42 confidence interval-CI (0.36,0.73), p = 0.0002). SOX2 was associated with significantly longer survival independent of histology in multivariate analysis (hazards ratio-HR = 0.429 confidence interval-CI (0.295, 0.663), p = <0.001).

**Conclusions:**

SOX2 is an independent positive prognostic marker in NSCLC. Increased SOX2 levels are more frequent in SCC than in ADC, but the association with better survival is independent from the histological subtype.

## Introduction

SOX2 belongs to the SRY-related HMG-box (SOX) family of embryonic developmental transcription factors [1]. SOX2 plays a critical role in lineage determination and embryonic development of the respiratory tract and the central nervous system [2], [3]. During development SOX2 is mainly expressed in the central/non-branching airways and appears to play a critical role in maintaining the stem cell-like phenotype in cancer cells [2], [4], [5]. SOX2 is amplified and overexpressed in several malignancies including lung, head and neck, esophageal, breast, gastric, and colon carcinomas [6]–[11]. Moreover, SOX2 expression is associated with aggressive phenotype and poor prognosis in several tumor types [12]–[16]. In lung neoplasms SOX2 is frequently upregulated and its gene amplification correlates with protein overexpression in NSCLC [4], [17]–[20]. SOX2 amplification and overexpression is more common in squamous cell carcinomas (SCC) than in lung adenocarcinomas (ADC) [4] and the prognostic impact of SOX2 overexpression in NSCLC appears to be dependent on the histologic subtype [17], [18]. Indeed. SOX2 amplification and overexpression were recently reported to be associated with better outcome in SCC [17], but with poor outcome in early stage lung ADC (n = 104) [18] The latter findings support the notion that SOX2 overexpression serves as positive prognostic indicator only in lung SCC and points towards a complex and dissimilar role of this transcription factor in the biology of the two major lung cancer subtypes. However and to our knowledge, these findings have not been clearly reproduced by other groups and validated in independent lung cancer cohorts [17] In addition, most of the studies evaluating SOX2 protein in lung tumors have used qualitative chromogenic immunohistochemistry with various antibodies and diverse scoring criteria.

In this study we investigate the prognostic role of SOX2 using automated quantitative immunofluorescence (QIF) in two independent lung cancer cohorts and analyzed the relationship between SOX2 levels and the main clinicopathologic features of patients with lung cancer. Our results show that increased tumor SOX2 levels predict better outcome in NSCLC and the effect is independent of histologic type.

## Methods

### Patient cohorts and Tissue Microarrays

Primary NSCLC tumor in the form of formalin-fixed paraffin-embedded tissue from patients at Yale University/New Haven Hospital between January 1980 and October 2003 were obtained from the Yale Pathology Tissue Services. The study was approved by the Human studies committee at Yale University. The data were analyzed anonymously from preexisting patient databases and hence exempt from consent by the human studies committee. In addition to our institutional cohort we assessed an independent cohort of 340 patients with NSCLC diagnosed between 1991 and 2001, obtained from the Sotiria General Hospital and Patras University General Hospital in Greece. In the Yale University lung cancer cohort, the median age of the patients was 66, with 147(48) % male and 160(52) % female patients. All the patients were treatment-naïve at the time of tumor resection or biopsy. The average follow-up period was 51 months (median 31 months Range (0,278)). In the Greek cohort the median age of the patients was 64, with 300(88) % male and 40(12) % female patients. The patient characteristics of this cohort are described in Table 1. The average follow-up period was 24 months (median 20 Range (0, 60)). The patient characteristics for both training cohort and validation cohort were described in Table 1.

**Table 1:** Clinical characteristics and SOX2 expression for the training and validation cohorts

Variables	Training Cohort (Greek Cohort)	Validation Cohort (Yale Cohort)	P value
Age(mean, sd)	62.32(9.04)	65.17(9.92)	0.0001
Gender (n,%)			
Female	40(11.76)	160(52.12)	<0.0001
Male	300(88.24)	147(47.88)	
Stage (n, %)			<0.0001
I&II	199(58.70)	234(73.58)	
III&IV	140(41.30)	84(26.42)	
Histology (n, %)			<0.0001
Adenocarcinoma	133(39.12)	212(63.28)	
Squamous cell carcinoma	167(49.12)	76(22.69)	
Other histologic subtypes	40(11.76)	47(14.03)	

Tissue specimens were prepared in a tissue microarray format: representative tumor areas were obtained from formalin-fixed, paraffin-embedded specimens of the primary tumor, and two 0.6-mm cores from each tumor block were arrayed in a recipient block. Control cell lines were formalin-fixed, paraffin-embedded and used as controls: HT29, MB435, BT474, SKBR3, H1299, A549, SW-480, H1666, H1335, MCF-7, HC15, A431, HCC2279, H2882, H1819, HC193, and H2126 were purchased from the American Type Culture Collection (Manassas, VA). Culture conditions and cell-line tissue microarray construction have been published in detail elsewhere [21].

### Antibodies and Immunohistochemistry

The arrays were deparaffinized in heat oven for 20 minutes at 55 C followed by serial xylene washes. They were rehydrated in graded alcohols, and subjected to antigen retrieval using citrate buffer (pH 6) PT module set for 20 min at 97 C. Slides were preincubated with 0.3% bovine serum albumin in 0.1 mol/L TBS for 30 min at room temperature. Slides were then incubated with a cocktail of the SOX2 primary antibody (rabbit monoclonal, clone D6D9; Cell Signaling Technology) diluted 1/100 and a mouse monoclonal antihuman cytokeratin antibody (clone AE1/AE3, M3515; Dako) diluted 1∶100 in bovine serum albumin/TBS overnight at 4°C. Following this the TMAs were incubated for 1 hour with Alexa 546-conjugated goat antimouse secondary antibody (A11003; Molecular Probes) diluted 1∶100 in rabbit EnVision reagent (K4003, Dako). Cyanine 5 (Cy5) directly conjugated to tyramide (FP1117; Perkin-Elmer) at a 1∶50 dilution was used as the fluorescent chromagen for target detection. Prolong mounting medium (ProLong Gold, P36931; Molecular Probes) with 4′,6-diamidino-2-phenylindole (DAPI) was used to stain nuclei in the histospot. Serial sections of a smaller “index” NSCLC array were stained aside both cohorts to confirm assay reproducibility.

**Figure 1:**
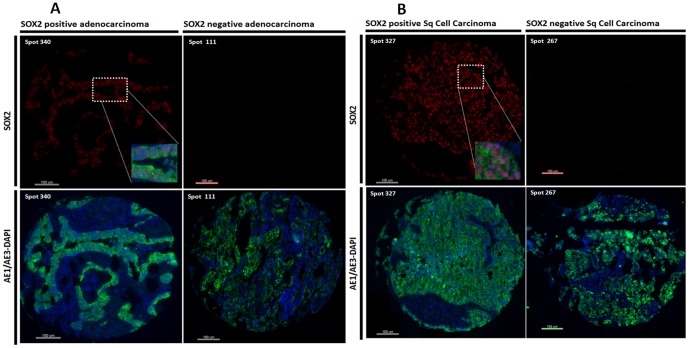
Representative immunofluorescence microphotographs of NSCLC samples (Fig. 1A: ADC and Fig. 1B:SCC) with high SOX2 levels and negative SOX2. The upper panels show SOX2 fluorescence (red channel) with characteristic nuclear staining pattern (left) and lack of SOX2 positivity (right). The lower panel shows cytokeratin immunoreactivity in the tumor compartment (AE1/AE3, green channel) and nuclear staining with DAPI (blue channel).

To evaluate for run to run variability both the Yale cohort and the Greek cohort were stained and analyzed on different days using the same conditions. The linear regressions (R^2^) between the two experiments were >0.7 for all the arrays (see figure 1). The specificity of the antibody was demonstrated with Western blot (using MCF7 and NTERA cell lysates as positive control and HELA, A431, BT20 and MB453 cell lysates as negative control).

### Automated quantitative immunofluorescence (QIF)

Automated quantitative analysis (AQUA™) enables objective measurement of protein concentration within user defined cellular compartments, as described in detail elsewhere [22]. Briefly, a series of monochromatic high-resolution images were captured using an epifluorescent microscope and an algorithm for image collection. For each histospot images were acquired using the signals detected from the SOX2-Cy5 channel, 4′,6-diamidino-2-phenylindole (DAPI) and cytokeratin-Alexa 546 channel. Tumor was distinguished from stromal and nonstromal elements by creating an epithelial tumor “mask” from the cytokeratin-Alexa 546 channel signals. A binary mask is created with each pixel being either “on” or “off” on the basis of an intensity threshold set by visual inspection of histospots. The AQUA score of SOX2 in each subcellular compartment was calculated by dividing the SOX2 compartment pixel intensities by the area of the compartment within which they were measured. (Figure 1) AQUA scores were normalized to the exposure time and bit depth at which the images were captured, allowing scores collected at different exposure times to be directly comparable.

### Statistical analysis

Using linear regressions, R^2^ greater than 0.4 was indicative of good inter- and intra-array reproducibility and thus the average values for SOX2 AQUA scores from duplicate samples were calculated and treated as independent continuous variables. X-tile software [23] was used to select the optimal SOX2 concentration cut point for the Greek lung cancer cohort (training set); this cut point was subsequently validated in the Yale University Lung Cancer cohort (validation set).

Patient characteristics were compared between training cohort and validation cohort by using t-test for continuous variables and chi-square test for categorical variables. The associations between SOX2 and characteristics variables were assessed by using t-test or analysis of variance (ANOVA). Overall survival (OS) functions between patients with high and low SOX2 expression were compared using log-rank test. Multivariate COX models were built to examine the effect of SOX2 on overall survival adjusted by the effect of age, gender, stage and histology.

#### Optimal cut point selection for SOX2 levels

Since this study was done using a method that provides continuous data, and we had no biological evidence to support a specific cut-point for cases stratification, we used a statistical-based strategy through X-tile software [23] to determine the optimal cutpoint of the SOX2 scores. Using this method every point of continuous variables is divided into two classes and a standard Monte Carlo simulation is performed to produce chi squared values which can be maximized to find the optimal cut point. Since the optimal separation in survival was obtained using a score of 193 in the Greek cohort, a second independent cohort was required for cutoff validation. Of note, the X-tile generated cutpoint also is the signal detection threshold for the assay in both the cohorts. Both the cohorts were stained in the same experiment using similar conditions and hence the scores were directly comparable without needing normalization.

## Results

### Assay validation and reproducibility in assessment of SOX2 measurements

**Figure 2:**
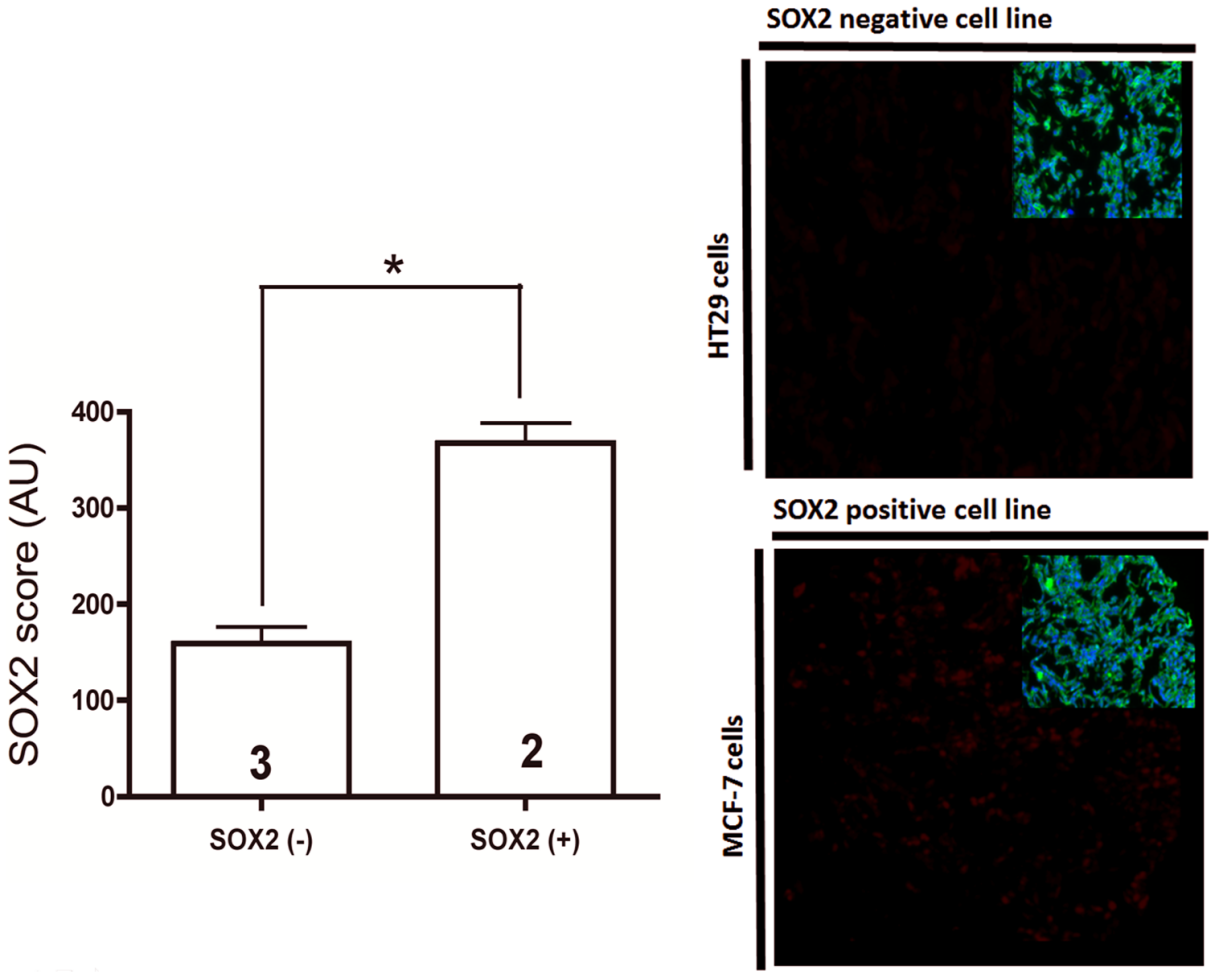
Validation of the assay using tumor cell lines with know SOX2 expression (MCF7 and BT474). SOX2 positive cell lines (MCF7 and BT474) show significantly higher SOX2 AQUA scores compared to cells with known low SOX2 expression (MB435, HT29 and A431 cell lines) (*P* value = <0.01). Representative fluorescence microphotographs of SOX2 immunoreactivity (red fluorescence channel) in MCF7 and HT29 cells. Insets shows cytokeratin immunostain(AE1/AE3, green channel) and nuclear DAPI fluorescence (blue channel). in the corresponding TMA spots.

To validate the QIF-based assay in FFPE specimens, cultured cell lines with known SOX2 expression were included together with tumor samples into TMAs, serially sectioned and stained. As expected, SOX2 signal was identified only in the tumor (CK-positive) compartment of lung carcinoma samples and showed a predominant nuclear staining pattern (Figure 1). Cell lines with known elevated SOX2 levels (MCF7 and BT474 [24]) showed higher SOX2 AQUA scores than the negative control cells with known low SOX2 expression (MB435, HT29 and A431, **p<0.01**, Figure 2).Of note, the AQUA scores obtained in the cell lines were considerably lower than in the SOX2-positive tumors.

**Figure 3:**
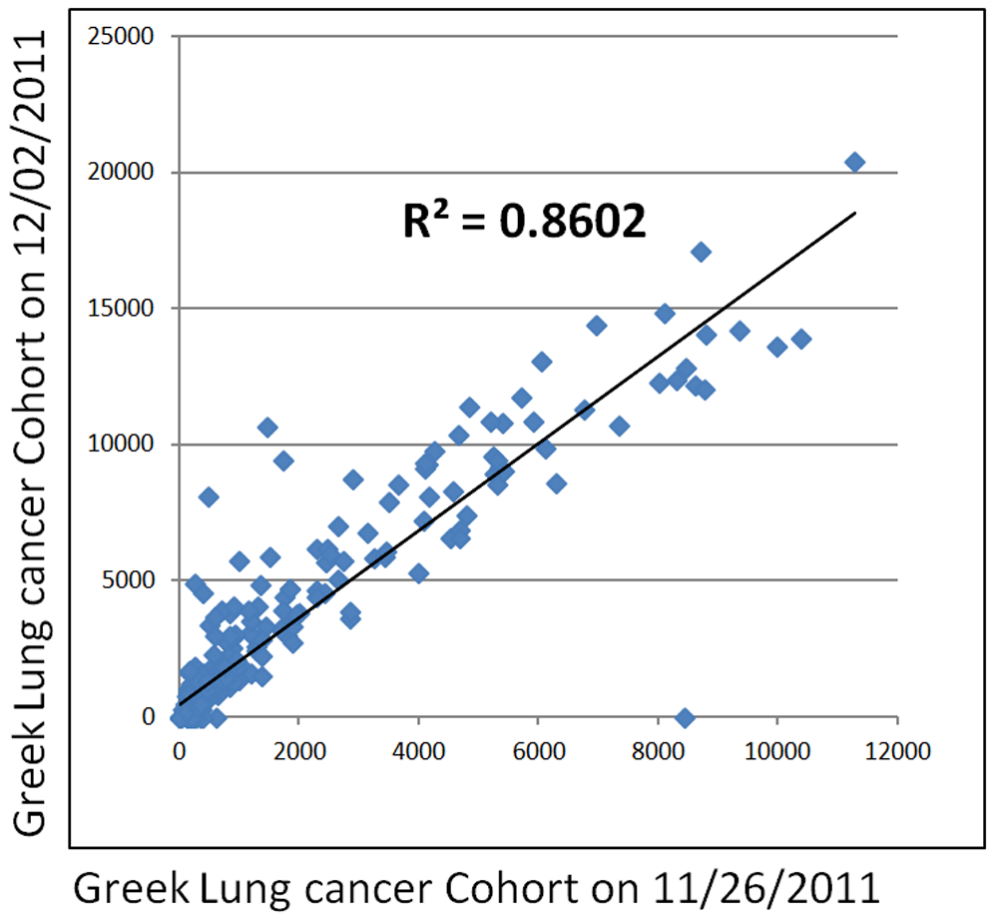
Inter-experiment reproducibility of SOX2 AQUA scores between two serial sections of the tissue microarray.

The levels of SOX2 in staining from serial sections of the lung cohort arrays showed a high linear regression coefficient (R^2^ = 0.86, Figure 3), indicating assay reproducibility. Intra-array reproducibility was also demonstrated by comparison of scores in spots present in 2 fold redundancy (R^2^ = 0.7158, not shown).

### Relationship between SOX2 levels and clinic-pathological variables

A description of the two lung cohorts is presented in Table 1.In the Greek cohort (training set), SOX2 levels were measured in 340 lung carcinomas, including 167 (49.12%) SCC,133 (39.12%) ADC, and 40 (11.76%) cases corresponding to other carcinoma subtypes. In the Yale cohort (validation set), SOX2 was measured in 335 lung tumors, including 76(22.69%) SCC, 212(63.28%) ADC and 47(14.03%) other histotypes.

The associations between SOX2 levels and the main clinical and pathology variables of the cohorts are presented in Table 2. In both lung cohorts SOX2 levels were ∼4 fold higher in patients with SCC as compared to ADC (p value<0.0001). In the Yale cohort only, SOX2 expression was higher in men than in female patients (p value 0.002). The overall SOX2 scores were lower in the Yale cohort than in the Greek array, possibly due to the considerable lower proportion of SCC in the former. No significant associations were noted with age or disease stage at diagnosis.

**Table 2:** Association between SOX2 and clinical characteristics.

	SOX2 in Training Cohort(Greek Cohort)	P value	SOX2 in Validation Cohort(Yale Cohort)	P value
Age		0.2439		0.9885
<70	1221.03(2072.81)		770.88(1456.02)	
> = 70	1550.39(2418.03)		773.20(1149.87)	
Gender(mean,sd)		0.8584		0.0022
Female	1351.96(2529.43)		519.10(791.17)	
Male	1286.98(2103.27)		1031.23(1739.55)	
Stage(mean, sd)		0.7999		0.2973
I&II	1322.84(2298.55)		833.55(1457.72)	
III&IV	1261.27(1939.51		640.90(1026.38)	
Histology(mean,sd)		<0.0001		<0.0001
Adenocarcinoma	454.96(792.94)		433.48(619.49)	
Squamous cell carcinoma	2032.35(2599.57)		1698.44(2230.20)	
Other subtypes	911.58(2071.85)		591.98(723.48)	

### Difference in the overall survival (OS) between patients with high and low SOX2 levels in the Greek cohort

**Figure 4:**
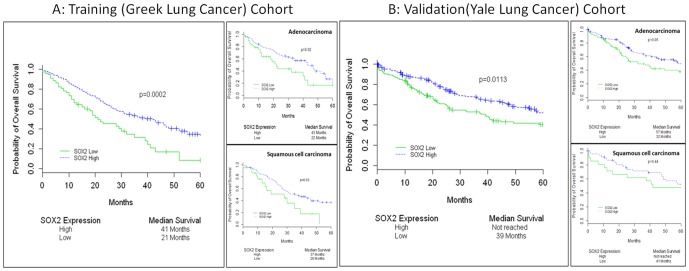
Correlation of SOX2 with overall survival: Survival curves in the training (4A) and validation (4B) datasets based on cohort division by the optimal cutpoint generated from the Greek Lung Cancer Cohort (training set). Panel A represents overall cohort and panels B and C represent survival in adenocarcinoma and squamous cell carcinoma, respectively.

In the Greek cohort, NSCLC patients with high SOX2 tumor levels (n = 245, scores >193) had longer overall survival than subjects with low tumor SOX2 (Figure 4, median survival 42 vs. 21 months; log rank p = 0.0002,). Moreover, high SOX2 levels resulted in 48% risk reduction in NSCLC patients at Cox proportional univariate analysis (hazards ratio-HR = 0.52 confidence interval-CI (0.36,0.73), p = 0.0002).

### Validation of the cutpoint and results in the Yale cohort

The cutpoint generated from the training set was applied in the validation cohort after normalization of scores to assess the potential of SOX2 to predict survival. In the Yale cohort, cases with high tumor SOX2 levels (n = 173) showed higher median survival compared to the low SOX2 expressers (Figure 4, median survival, not reached vs. 39 months respectively, log rank p = 0.0113). In addition, elevated tumor SOX2 resulted in 36% reduction in risk for all NSCLC patients at Cox univariate analysis (hazards ratio-HR = 0.64 confidence interval-CI (0.46,0.91), p = 0.01). Taken together, these results validated the X-tile defined cutpoint for SOX2 score stratification in lung carcinoma samples.

### Assessment of the independent potential of SOX2 to predict survival

Using multivariate cox proportional hazards regression model, risk estimates related to survival for gender, stage, histological type and SOX2 levels were estimated (Table 3); cases with missing values were excluded. As in the Greek training cohort, SOX2 is an independent prognostic marker in NSCLC patients in the Yale cohort (hazards ratio-HR = 0.429 confidence interval-CI (0.295, 0.663), p = <0.001).

**Table 3:** Mulivariate table with subgroup analysis for squamous cell carcinoma and non-squamous cell carcinoma.

Training cohort (Greek Cohort)						
	All Patients		Squamous Cell Ca		Non Squamous	
Characteristic	HR(95%)	P value	HR(95%)	P value	HR(95%)	P value
Age	1.022(1.003,1.043)	0.0246	1.029(0.999,1.06)	0.0619	1.015(0.987,1.04)	0.2952
Gender Female vs. Male	1.342(0.833,2.163)	0.2270	1.409(0.693,2.86)	0.3441	1.232(0.627,2.42)	0.5451
Stage I&II vs. III&IV	0.404(0.290,0.561)	<0.0001	0.467(0.288,0.75)	0.0021	0.356(0.224,0.56)	<0.0001
Histology Squamous vs. other	1.059(0.760,1.475)	0.7368	na		na	na
SOX2 High vs. low	0.429(0.295,0.663)	<0.0001	0.406(0.220,0.74)	0.0038	0.461(0.287,0.73)	0.0013
Validation cohort (Yale Cohort)						
Age	1.012(0.991,1.033)	0.2635	0.977(0.934,1.022)	0.3154	1.019(0.995,1.043)	0.1230
Gender Female vs. Male	0.717(0.484,1.060)	0.0952	2.128(0.870,5.206)	0.0982	0.613(0.399,0.943)	0.0259
Stage I&II vs III&IV.	0.426(0.284,0.637)	<0.0001	0.214(0.083,0.549)	0.0013	0.456(0.290,0.719)	0.0007
Histology Squamous vs. other	0.758(0.474,1.212)	0.2477	na		na	na
SOX2 High vs. low	0.734(0.502,1.075)	0.1121	0.985(0.419,2.320)	0.9733	0.702(0.456,1.080)	0.1075

## Discussion

SOX2 expression has been correlated with aggressive phenotype and poor prognosis in several tumor types [7], [8], [10], [15]. However, in patients with squamous cell NSCLC SOX2 expression appears to predict good outcomes [17]. In patients with stage1 lung adenocarcinoma, Sholl et al found the SOX2 expression predicts poor survival [18]. These findings have not been validated and are limited by small sample size and semi-quantitative methods of detection of SOX2 expression.

In this study, we measured the tissue SOX2 levels in two large independent cohorts of patients with NSCLC. We found that SOX2 levels are significantly higher in lung SCC relative to ADC. In addition, high SOX2 predicts better outcome in patients with NSCLC. In SCC subtype the association with survival was significant in the Greek cohort and had a trend towards better survival in the Yale cohort. This is likely because of small number of patients with SCC in the Yale cohort, reflecting the distribution of NSCLC histological subtypes in the US population.

These findings are consistent with previous findings from Bass et al and Wilbertz et al. [17], [19] and confirm the association between SOX2 and SCC subtype; and the independent positive prognostic value of SOX2 in NSCLC. Interestingly, a high SOX2 level in ADC in both studied lung cohorts was also associated with longer survival. This is contrary to the findings reported by Sholl et al. where SOX2 expression correlated with poor outcomes in patients with stage I adenocarcinomas [18]. Wilbertz et al evaluated SOX2 expression by IHC in two cohorts of patients comprising of 315 and 240 patients. They reported no association between SOX2 protein expression and survival in lung adenocarcinomas in either cohort. However, patients with low-level of SOX2 amplifications had poor survival in one of the cohorts (p = 0.009). SOX2 expression was associated with higher tumor grade [17]. The findings from Sholl et al. however were in a relatively small cohort (n = 104) and included only early stage lung ADC. In addition, both studies by Scholl and Wilbertz used qualitative IHC for SOX2 detection and results were not validated in independent external cohorts. The aforementioned factors may, at least in part, account for the striking differences between the reported studies and our findings. Further work including larger series of lung ADC and comparable/multiple SOX2 detection assays will be required to clarify this apparent contradiction.

The mechanisms of regulation and functional role of SOX2 are not clearly understood. SOX2 levels correlate frequently with SOX2 gene amplification. However, SOX2 amplification is a less common event in adenocarcinoma relative to squamous cell carcinoma [4], [17]. There may be mechanisms other than SOX2 gene amplification driving increased SOX2 protein, particularly in lung adenocarcinomas. Despite the molecular differences and differences in the copy number changes of SOX2 in squamous cell carcinoma and adenocarcinoma, SOX2 expression is an independent predictor of prolonged survival in both squamous cell carcinoma and adenocarcinoma of the lung.

While these results show that SOX2 expression is associated with better outcome in both SCC and ADC, our study has a number of limitations. First, this is a retrospective study and should be interpreted with caution. A second limitation is that the work was performed on TMAs, which may underestimate the heterogeneity of SOX2. Furthermore, TMAs are not used as a standard diagnostic method. Previous studies in other tumor types have shown that TMAs can be representative of tumor samples although the level of redundancy required for adequate representativity appears to vary with the antigen being measured [25]. The level of heterogeneity for SOX2 has not yet been defined, but the observation of association with outcome in two unrelated cohorts increases our confidence in the use of TMAs for this study. In addition, the linear regression coefficient for the SOX2 measurements in samples studied in two fold redundancy within the Yale cohort further confirm this notion (R^2^ = 0.7158, not shown).

The measurement of SOX2 has potential to help risk stratification of NSCLC patients. While this work is hypothesis generating and the measurement of SOX2 in the clinic would be premature, this work reinforces the importance of SOX2 in NSCLC biology and raises the possibility of using this transcription factor in future NSCLC classification models.
